# Dynamic Orchestration of Brains and Instruments During Free Guitar Improvisation

**DOI:** 10.3389/fnint.2019.00050

**Published:** 2019-09-04

**Authors:** Viktor Müller, Ulman Lindenberger

**Affiliations:** ^1^Center for Lifespan Psychology, Max Planck Institute for Human Development, Berlin, Germany; ^2^Max Planck UCL Centre for Computational Psychiatry and Ageing Research, Berlin, Germany; ^3^Max Planck UCL Centre for Computational Psychiatry and Ageing Research, London, United Kingdom

**Keywords:** intra- and inter-brain coupling, brain-instrument coupling, graph-theoretical approach, EEG hyperscanning, phase synchronization, extended hyper-brain networks, social interaction

## Abstract

Playing music in ensemble requires enhanced sensorimotor coordination and the non-verbal communication of musicians that need to coordinate their actions precisely with those of others. As shown in our previous studies on guitar duets, and also on a guitar quartet, intra- and inter-brain synchronization plays an essential role during such interaction. At the same time, sensorimotor coordination as an essential part of this interaction requires being in sync with the auditory signals coming from the played instruments. In this study, using acoustic recordings of guitar playing and electroencephalographic (EEG) recordings of brain activity from guitarists playing in duet, we aimed to explore whether the musicians' brain activity synchronized with instrument sounds produced during guitar playing. To do so, we established an analytical method based on phase synchronization between time-frequency transformed guitar signals and raw EEG signals. Given phase synchronization, or coupling between guitar and brain signals, we constructed so-called *extended* hyper-brain networks comprising all possible interactions between two guitars and two brains. Applying a graph-theoretical approach to these networks assessed across time, we present dynamic changes of coupling strengths or dynamic orchestration of brains and instruments during free guitar improvisation for the first time. We also show that these dynamic network topology changes are oscillatory in nature and are characterized by specific spectral peaks, indicating the temporal structure in the synchronization patterns between guitars and brains. Moreover, extended hyper-brain networks exhibit specific modular organization varying in time, and binding each time, different parts of the network into the modules, which were mostly heterogeneous (i.e., comprising signals from different instruments and brains or parts of them). This suggests that the method capturing synchronization between instruments and brains when playing music provides crucial information about the underlying mechanisms. We conclude that this method may be an indispensable tool in the investigation of social interaction, music therapy, and rehabilitation dynamics.

## Introduction

As noted by D'Ausilio et al. (2015): “Group-level musical coordination can be considered as a microcosm of social interaction.” A recently emerging view in music neuroscience with regard to hyperscanning methods holds that playing music in groups is not only social and interactive (Keller et al., 2014; Acquadro et al., 2016; Chang et al., 2017), but that it requires strong inter-brain synchronization and specific hyper-brain network activity supporting interpersonal action coordination (Lindenberger et al., 2009; Sänger et al., 2011, 2012, 2013; Müller et al., 2013, 2018b). This hyper-brain network activity including both intra- and inter-brain synchronization is enhanced during periods of high demands on musical coordination and is accompanied by the emergence of so-called hyper-brain modules composed of nodes from two or more brains. It has also been shown that the topology of hyper-brain networks involving two (Sänger et al., 2012; Müller et al., 2013) or even four (Müller et al., 2018b) brains revealed small-world properties with high segregation and integration of brain function, and had a tendency to become more random at lower frequencies and more regular at higher frequencies. Moreover, this topology was characterized by a higher number of hub-connectors at the delta and theta frequency of brain signals during joint, as compared to solo guitar playing (Müller et al., 2013). Furthermore, different types of information flow—intra- vs. inter-modular—were found when playing guitar in quartet (Müller et al., 2018b). Nevertheless, little, if anything, is known about the interaction between brain processes or mechanisms implementing interpersonally coordinated behavior when playing music and the instruments used for music production.

In the current study, we attempt to close the conceptual gap between these important elements of musically coordinated behavior—music production and its neuronal implementation. Using acoustic recordings of guitar playing and electroencephalographic (EEG) recordings of the brain activity of guitarists playing guitar in duet, we tried to establish a method to investigate the couplings within and between all components of duet playing, i.e., the guitars and brains. All these couplings were then used to construct and to analyze a so-called *extended* hyper-brain network including two guitars and two brains and all connections within and between them. We describe different situations of connections between guitars and brains here, which together exhibit complex networks with network topology changing in time and reflecting the dynamic orchestration of brains and instruments in guitar duos.

## Materials and Methods

### Participants

Two pairs of professional guitarists participated in the study. In both pairs, the lead guitarist was always the same individual. The guitarists in the duo were not known to each other. All participants were right-handed and had played the guitar professionally for more than 5 years. The study was approved by the ethics committee of Max Planck Institute for Human Development (Berlin), and therefore performed in accordance with the ethical standards laid down in the 1964 Declaration of Helsinki. All subjects volunteered for this experiment and gave their written informed consent prior to their inclusion in the study.

### Experimental Procedure and Data Acquisition

During the experiment, the guitarists sat facing each other and freely improvised in duet for about 5–6 min. Before playing, the guitarists had the opportunity to briefly discuss the theme of their improvisation. Typically, one guitarist played a single-line melody or solo, while the other accompanied with chords or in another way. They also switched roles several times during the improvisation. Participants were instructed to avoid unnecessary movement in order to reduce movement artifacts.

Acoustic and EEG measurement took place in an acoustically and electromagnetically shielded cabin. EEG was simultaneously recorded from both members of the pair using two electrode caps with 64 Ag/AgCl electrodes placed according to the international 10–10 system, with the reference electrode placed at the right mastoid. For further analysis, we used 40 EEG channels for each subject. These channels or electrodes were distributed across the entire cortex, so that the information of the remaining electrodes would be rather redundant. Separate amplifiers with separate grounds were used for each individual, optically coupled to a computer. The vertical and horizontal electrooculogram (EOG) was also recorded to control for eye blinks and eye movements. Sampling rate was 5,000 Hz. The anti-aliasing filter was set to 1,000 Hz. A notch filter was used to suppress 50 Hz noise. EEG recordings were re-referenced offline to an average of the left and right mastoids and then filtered with a band pass ranging from 0.5 to 70 Hz. Eye movement correction was accomplished by independent component analysis (Vigário, 1997). Thereafter, artifacts from head and body movements were rejected by visual inspection. Spontaneous EEG activity was resampled at 250 Hz and divided into artifact-free 10-s epochs. In all, 10 artifact-free 10-s epochs were used for coupling and network analyses.

The sounds of the guitars were recorded through two microphones (i.e., one for each guitar) on two EEG channels, simultaneously with the EEG recordings. These two sound signals were divided into corresponding 10-s epochs without resampling. In addition, video and sound were recorded using Video Recorder Software (Brain Products, Munich, Germany) synchronized with EEG data acquisition.

### Data Analysis

**Figure 1:**
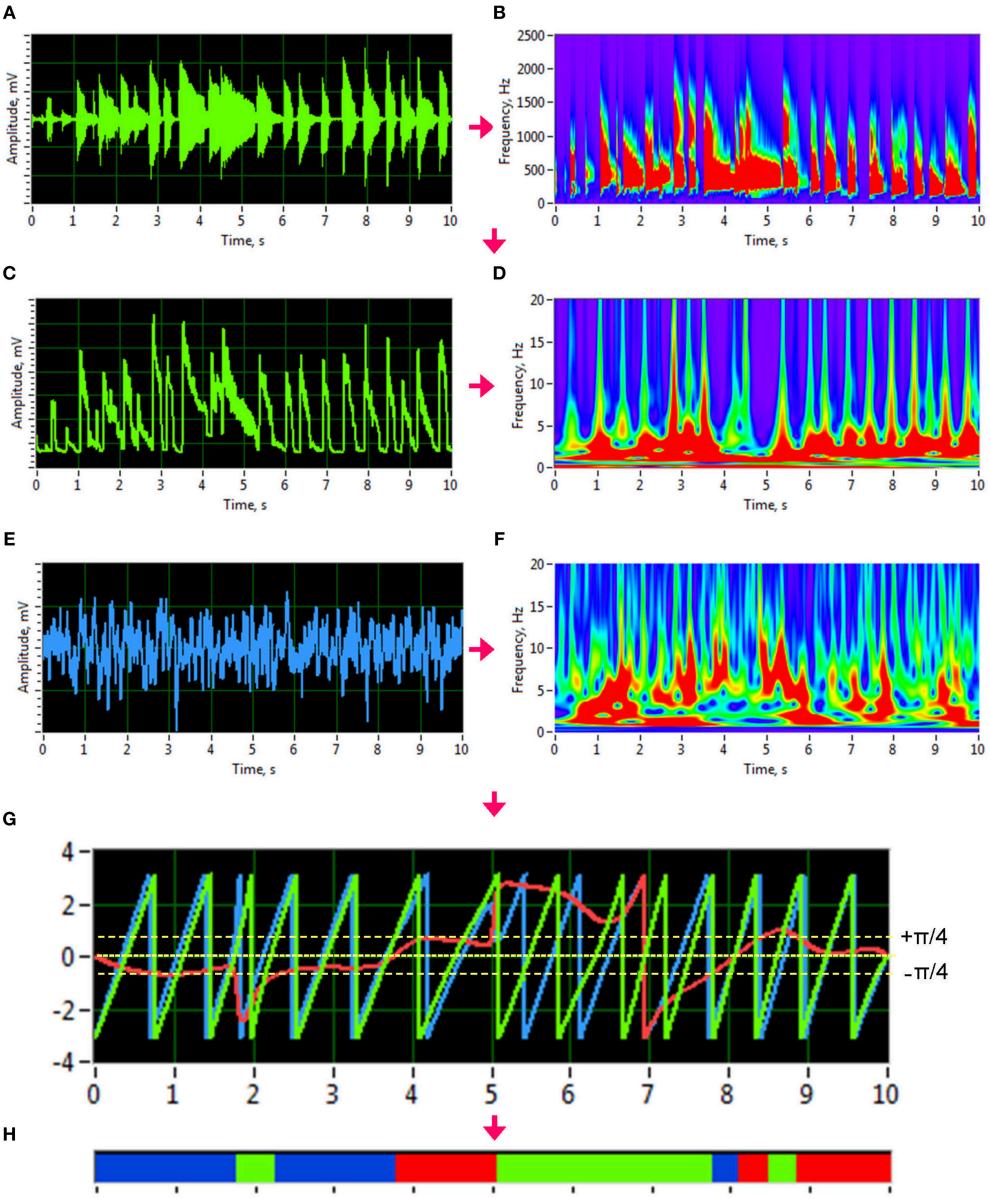
Transformation of guitar signals and calculation of the Integrative Coupling Index (ICI). **(A)** Raw signal of the guitar recording. **(B)** Wavelet transform of the guitar recording in time-frequency domain. **(C)** New guitar signal derived from the wavelet-transformed signal by averaging the wavelet power across the frequency bins of interest. The whole range between 50 and 2,000 Hz is presented here. **(D)** Wavelet transform of the new guitar signal in the time-frequency domain used for calculation of the *ICI*. **(E)** Raw EEG signal. **(F)** Wavelet transform of the EEG signal in time-frequency domain. **(G)** Time course of instantaneous phases from two signals and their phase difference. Phase of the guitar signal = green curve; phase of the EEG signal = blue curve; phase difference (Δφ) between the two signals = red curve). **(H)** Coding of the phase difference (–p/4 < Δφ < 0: blue stripes; 0 < Δφ < +p/4: red stripes; Δφ < –p/4 or Δφ > +p/4: green stripes = non-synchronization).

To investigate phase synchronization or coupling between the signals, we first normalized the high-frequency auditory signals and applied an analytic Morlet wavelet transform (Figures 1A,B) to calculate the power or amplitude within the four different frequency ranges: low (50–250 Hz), middle (250–500 Hz), high (500–2,000 Hz), and whole range (50–2,000 Hz). By averaging the amplitude within these four frequency ranges, we generated low-frequency time series that varied in a frequency range comparable to the EEG time series (see Figure 1 for details). To investigate phase coupling between the given signals in a directed and frequency-resolved manner, we calculated the Integrative Coupling Index (*ICI*) described elsewhere (Müller and Lindenberger, 2011; Müller et al., 2013). To do so, we applied an analytic complex-valued Morlet wavelet transform to both the generated auditory (Figures 1C,D) and EEG time series (Figures 1E,F), and computed the instantaneous phases for four frequencies of interest or frequency components (FC): 1.25, 2.5, 5, and 10 Hz (FC1, FC2, FC3, and FC4, respectively). The complex mother Morlet wavelet, also called Gabor wavelet, has a Gaussian shape around its central frequency *f* :

(1) $$\begin{array}{r} w\left(t,f\right)=\frac{1}{\sqrt[{4}]{\sigma^{2}\pi }}\text{exp }\left(-\frac{t^{2}}{2\sigma^{2}}+\frac{3}{2}\pi jft\right),j=\sqrt{-1} \end{array}$$

where σ is the standard deviation of the Gaussian envelope of the mother wavelet. The frequency resolution of the wavelet transform was fixed at 0.125 Hz, and time resolution was fixed at 4 ms.

In order to identify the phase relations between any two channels (X and Y), the instantaneous phase difference ΔΦ_*XY*_(*t, f*) was computed from the wavelet coefficients for all possible electrode and transformed acoustic signal pairs:

(2) $$\begin{array}{r} \Delta \Phi_{XY}^{k}\left(t,f\right)=\text{mod }\left(\Phi_{X}^{k}\left(t,f\right)-\Phi_{Y}^{k}\left(t,f\right),2\pi \right) \end{array}$$

With instantaneous phases of the two signals across *k* data points in the segment: $\Phi_{X}^{k}\left(t,f\right)=arg\;\{z_{X}^{k}\left(t,f\right)\}$ and $\Phi_{Y}^{k}\left(t,f\right)=arg\;\{z_{Y}^{k}\left(t,f\right)\}$.

Given the estimates of the phase difference between the two signals, it is possible to ascertain how long the phase difference remains stable in defined phase angle boundaries by counting the number of points that are phase-locked within a defined time window. In accordance with the procedure described by Müller and Lindenberger (2011) (cf. Müller et al., 2013), we divided the range between –π/4 and +π/4 into two ranges and distinguished between positive and negative deviations from phase zero (Figure 1G). We marked negative deviations in the range between –π/4 and 0 in blue (coded with “−1”) and the positive deviations in the range between 0 and +π/4 in red (coded with “+1”). Phase difference values beyond these ranges were marked in green (coded with “0”) and represent non-synchronization (Figure 1H). For two channels X and Y, a blue stripe in the diagram would mean that the phase of channel Y precedes the phase of channel X, while a red stripe would mean that the phase of channel X precedes the phase of channel Y. We then counted the number of phase-locked data points, for both ranges separately. Before counting, successive points in the defined range (between –π/4 and +π/4) with a time interval shorter than a period (*T*_*i*_ = 1/*f*_*i*_) of the corresponding oscillation at the given frequency *f*_*i*_ were discarded from the analysis. This cleaning procedure effectively eliminated instances of accidental synchronization. On the basis of this counting, we obtained four synchronization indices: (1) the *Positive Coupling Index, PCI*, or the relative number of phase-locked points in the positive range (between 0 and π/4); (2) the *Negative Coupling Index, NCI*, or the relative number of phase-locked points in the negative range (between –π/4 and 0); (3) the *Absolute Coupling Index, ACI*, or the relative number of phase-locked points in the positive and negative range (i.e., between –π/4 and +π/4); and (4) the *Integrative Coupling Index, ICI*, calculated by the formula:

(3) $$\begin{array}{r} ICI=\frac{PCI+ACI}{2\cdot ACI}\cdot \sqrt{PCI} \end{array}$$

The *ICI* is equal to 1 when all points are phase-locked and positive; if all phase-locked points are negative or are out of range, the *ICI* will approach 0. Thus, the *ICI* measure ranges from 0 to 1 and is asymmetric (*ICI*_XY_ ≠ *ICI*_YX_), indicating the relative extent of the positive shift in phase difference between two signals. We restrict the description of our study results to the *ICI* measure, which is the most informative index due to its directionality. For dynamic representation of coupling within the 10-s segments, we calculated phase coupling using moving time windows of 2,000 ms width and 80 ms time delay. Overall, within a segment of 10-s duration, coupling measures across 101 time windows were collected by this shifting procedure. The Matlab code to calculate the *ICI* measure from the phase difference between the two signals can be found in the Supplementary Material.

### Network Construction and Calculation of Strengths

**Figure 2:**
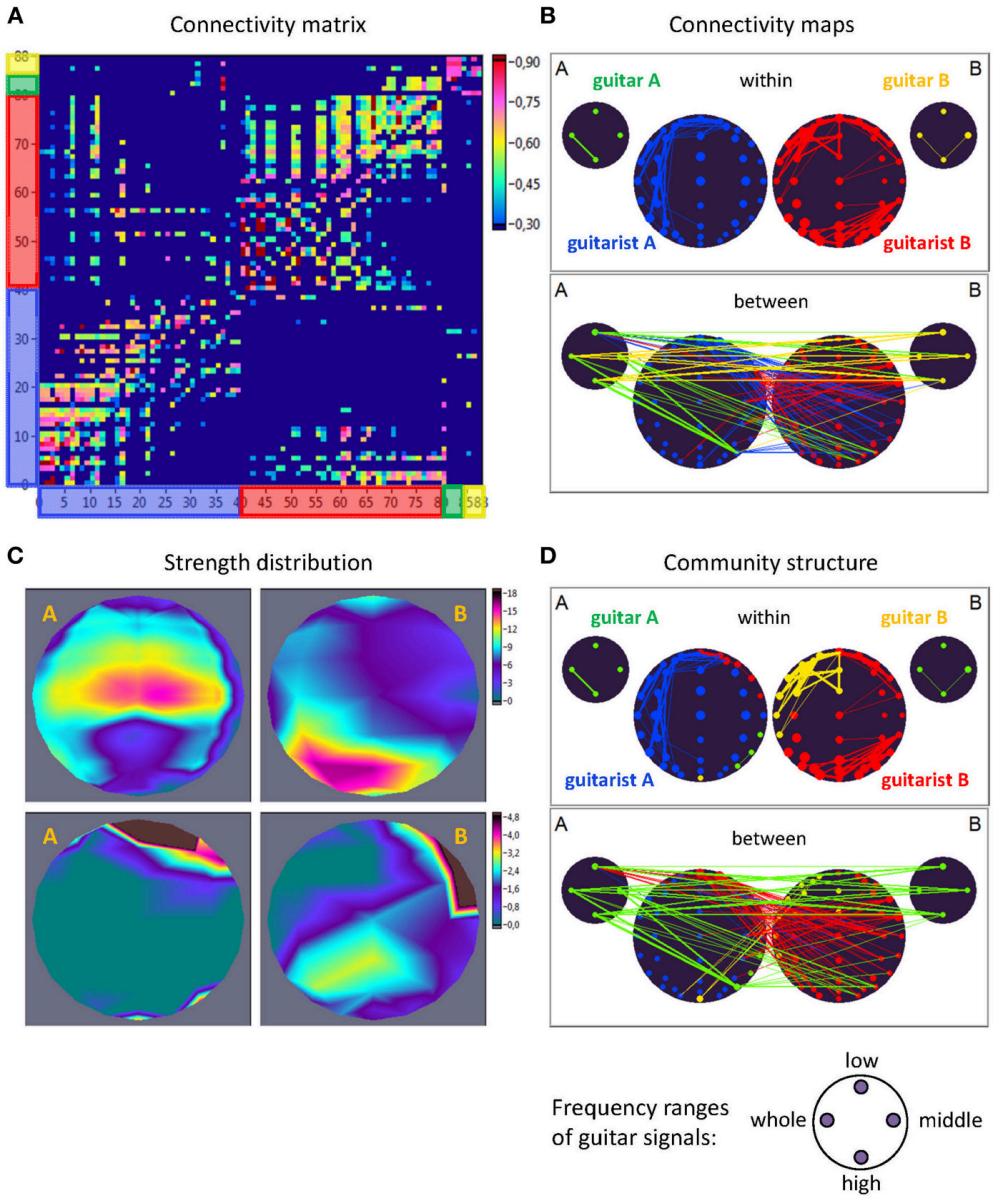
Extended hyper-brain network. **(A)** Connectivity matrix. The network consisting of 88 nodes and 7,656 edges in total includes 40 nodes (electrodes) of guitarist A's brain, 40 nodes of guitarist B's brain, four nodes of guitar A, and 4 nodes of guitar B. The four nodes in the guitars display four different frequency ranges used during signal transformation. The connection strength in the matrix is displayed by colors ranging from dark blue (threshold connectivity values) to dark red (high connectivity values). **(B)** Connectivity maps. The upper panel represents the connectivity within the guitars and brains, and the lower panel represents the connectivity between the guitars and brains. The strength of the nodes (sum of all out-going connections) is coded by circle size, and the strength of edges is coded by the width of the line. The different parts of the network are color-coded: guitar A, green; guitar B, yellow; guitarist A's brain, blue; guitarist B's brain, red. **(C)** Topological distribution of the strengths. The upper two maps represent the topological distribution of the out-strengths within the brains of the two guitarists, and the lower ones display the topological distribution of the out-strengths going from guitarist A's brain to guitarist B's brain (left) and vice versa (right). **(D)** Community structure of the network. The connectivity maps are presented as in **(B)** but nodes and corresponding edges are color-coded according to module affiliation. Note that most of the modules are heterogeneous, comprising different guitars and brains, with exception of the blue module, which is located within the brain of guitarist A.

For construction of the *extended* hyper-brain network, we calculated the *ICI* between all electrode pairs within and between the brains as well as within and between the guitars using the four different frequency components corresponding to the four ranges described above (low, middle, high, and whole range). In addition, we calculated the *ICI* between the brains and the guitars. Given all these couplings, we finally constructed an extended hyper-brain network comprising 88 nodes (40 + 40 + 4 + 4) and 7,656 edges (all possible couplings between the nodes) for each FC (1.25, 2.5, 5, and 10 Hz) and each time window. Figure 2 shows an example of an extended hyper-brain network in the form of the connectivity matrix (Figure 2A) and connectivity maps (Figure 2B).

In order to determine the network properties, we set the cost level (ratio of the number of actual connections divided by the maximum possible number of connections in the network) to 25%, which makes it possible to investigate sparse networks with the same number of edges at different FCs and time windows. The connectivity threshold was always higher than the significance level determined by the surrogate data procedure (i.e., networks at this cost or sparsity level always included significant connections). This allowed more accurate examination of the network topology in the different duos and playing conditions. Surrogate data were created in two ways: (1) by random permutations of the time series under consideration, and (2) by phase permutation of the given time series. The phase permutation procedure involved: (a) computing the amplitude and phase spectrum of a real signal using a Fourier transformation; (b) phase shuffling, whereby the phase values of the original spectrum are used in random order and the sorted values of the surrogate sequence are replaced by the corresponding sorted values of the reference sequence; and (c) inverse Fourier transformation back to the time domain. In this way, the real and the surrogate data retain the same power spectrum but a different time course due to phase shuffling. For both surrogate data procedures, 10,000 permutations were used.

As *ICI* is a directed weighted measure, we obtained the nodes' in- and out-strengths, with the in-strength defined as the sum of weights of all incoming connections $(w_{ji}),S_{in}=\sum_{j\in N}w_{ji}$, and the out-strength as the sum of weights of all outgoing connections $(w_{ij}),S_{out}=\sum_{j\in N}w_{ij}$. For representation of network dynamics, we used out-strengths (*S*_*out*_) that were first determined for each node separately, and then grouped and summed for: (a) the out-strength going from each node of the guitar (A and B, separately) to the both brains of the guitarists, (b) the out-strength going from guitar A to guitar B and vice versa, (c) the coupling within the brains for each guitarist (A and B) separately, (d) the coupling between the brains with the out-strength going from guitarist A's brain to guitarist B's brain and vice versa, (e) the hyper-brain network comprising electrodes or nodes from two guitarists' brains.

As shown in Figure 2, the out-strengths are visualized in two different ways. First, in the connectivity maps (Figure 2B), the strengths are coded by the size of the nodes. Second, we present the topological distribution of strength, as displayed in Figure 2C.

### Modularity Analysis and Modular Organization of the Extended Hyper-Brain Network

To further investigate the modular organization of the networks, community structures and the modularity index (*Q*) were determined. For this calculation, we used the modularity optimization method for directed graphs that is implemented in the Brain Connectivity Toolbox (Rubinov and Sporns, 2010). The optimal community structure is a subdivision of the network into non-overlapping groups of nodes or communities in a way that maximizes the number of within-module edges and minimizes the number of between-module edges. *Q* is a statistic that quantifies the degree to which the network may be subdivided into such clearly delineated groups or modules. For directed networks, this is given by the formula (Leicht and Newman, 2008):

(4) $$\begin{array}{r} Q^{\to }=\frac{1}{l}\underset{i,j\in N}{\sum }\left[a_{ij}-\frac{k_{i}^{in}k_{i}^{out}}{l}\right]\cdot \delta_{m_{i},m_{j},} \end{array}$$

where *l* = ∑_*ij*_*a*_*ij*_ is the number of edges in the graph, and *a*_*ij*_ is defined to be 1 if there is an edge from *j* to *i*, and 0 otherwise, $k_{i}^{in}$ and $k_{i}^{out}$ are the in- and out-degrees of the node *i*, and δ_*m*_*i*_, *m*_*j*__ is the Kronecker delta, where δ_*m*_*i*_, *m*_*j*__ = 1 if *m*_*i*_ = *m*_*j*_, and 0 otherwise. High modularity values indicate strong separation of the nodes into modules. For this analysis, the extended hyper-brain network including all the nodes of the instruments and brains was used. Due to the fact that within-module edges are maximized by this partition procedure, the module or network community comprises those nodes with the strongest connections, which can represent different parts of the brains or instruments. One can assume that these modules or communities must have a specific functional meaning (Müller et al., 2018b). The community structures are presented as connectivity maps, where different modules are coded by color (see Figure 2D for an example).

## Results

**Figure 3:**
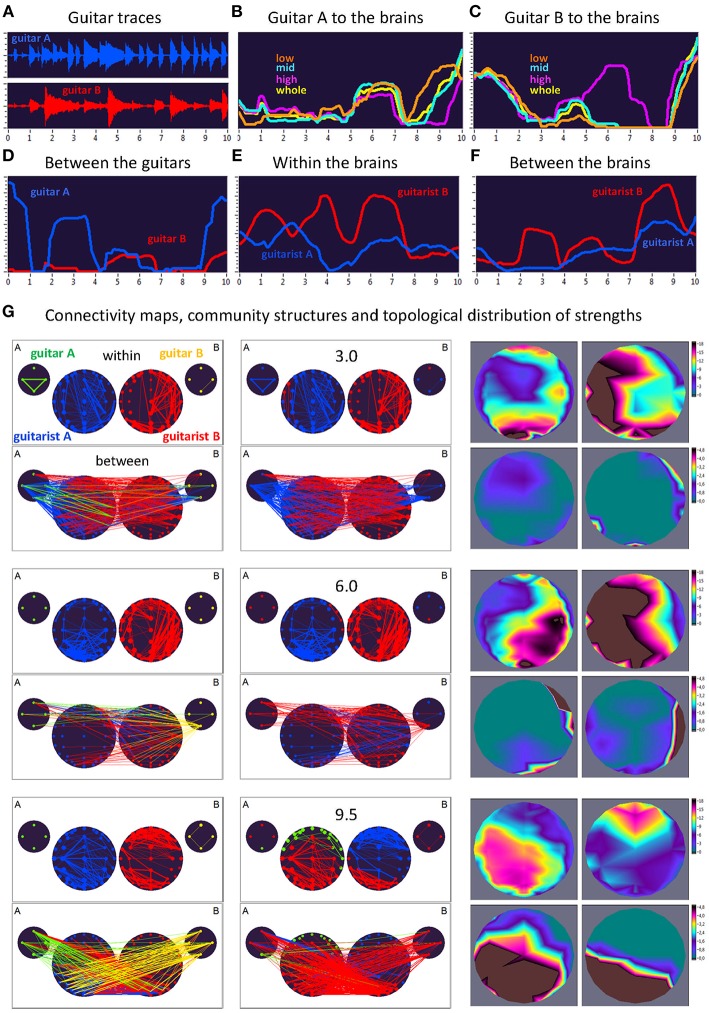
Dynamic changes of strengths during a 10-s improvisation period for FC1 (1.25 Hz) in duo 1. **(A)** Guitar traces obtained by microphone recording: guitar A, blue; guitar B, red. **(B)** Dynamic changes of coupling strengths going from guitar A to the brains of both guitarists for each of the four frequency ranges of the guitar signal, which are indicated by color: low range, brown; middle range, cyan; high range, purple; whole range, yellow. **(C)** Dynamic changes of coupling strengths going from guitar A to the brains of both guitarists for each of the four frequency ranges of the guitar signal, which are indicated by the same colors as in **(B)**. **(D)** Dynamic changes of coupling strengths going from guitar A to the guitar B (blue) and vice versa (red). **(E)** Dynamic changes of coupling strengths within the brains of each of the two guitarists. **(F)** Dynamic changes of coupling strengths going from guitarist A's brain to guitarist B's brain (blue) and vice versa (red). **(G)** Brain connectivity maps, community structures, and topological distribution of coupling strengths. See Figure 2 for explanations.

**Figure 4:**
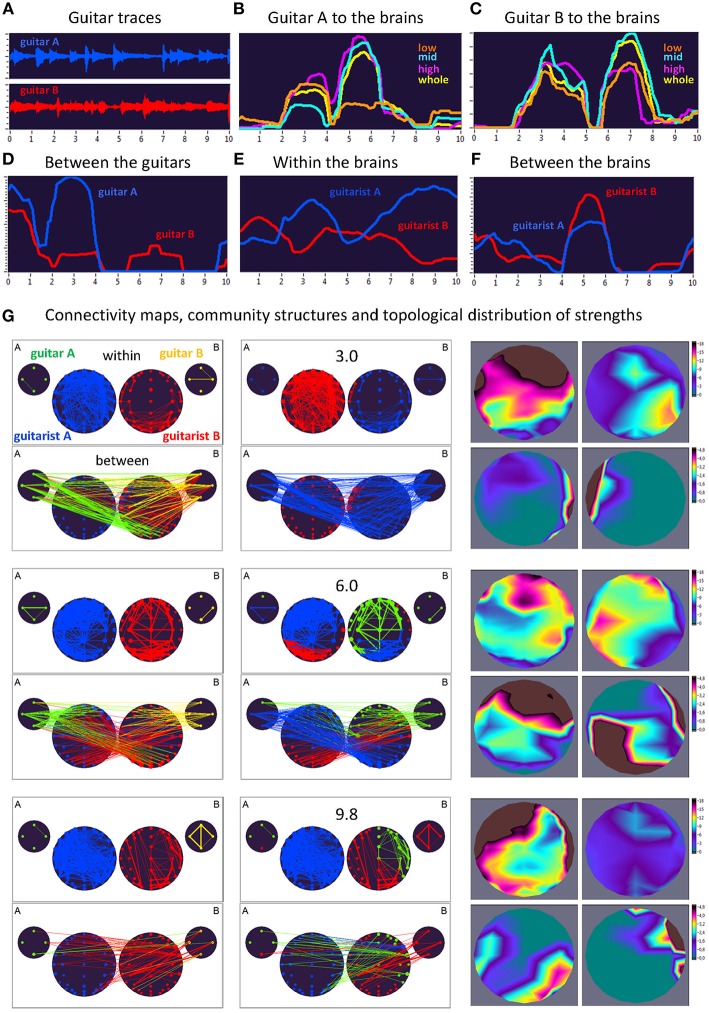
Dynamic changes of strengths during a 10-s improvisation period for FC1 (1.25 Hz) in duo 2. **(A)** Guitar traces obtained by microphone recording: guitar A, blue; guitar B, red. **(B)** Dynamic changes of coupling strengths going from guitar A to the brains of both guitarists for each of the four frequency ranges of the guitar signal, which are indicated by color: low range, brown; middle range, cyan; high range, purple; whole range, yellow. **(C)** Dynamic changes of coupling strengths going from guitar A to the brains of both guitarists for each of the four frequency ranges of the guitar signal, which are indicated by the same colors as in **(B)**. **(D)** Dynamic changes of coupling strengths going from guitar A to guitar B (blue) and vice versa (red). **(E)** Dynamic changes of coupling strengths within the brains of each of the two guitarists. **(F)** Dynamic changes of coupling strengths going from guitarist A's brain to guitarist B's brain (blue) and vice versa (red). **(G)** Brain connectivity maps, community structures, and topological distribution of coupling strengths. See Figure 2 for explanations.

Here, we exemplarily present data on the coupling between the instrument sounds and the brains of two guitarists when improvising freely in duets. Figures 3, 4 display the traces of guitars A and B (panel A), dynamic changes of coupling strengths going from guitar A (panel B) and guitar B (panel C) to the brains of both guitarists for each of the four frequency components of the guitar signals, dynamic changes of coupling strengths going from guitar A to the guitar B and vice versa (panel D), dynamic changes of coupling strengths within the brains of each of the two guitarists (panel E), dynamic changes of coupling strengths going from the guitarist A's brain to the guitarist B's brain and vice versa (panel F), and brain connectivity maps (left column) of the coupling within (upper map) and between (lower map) the brains, with extended hyper-brain community structures (middle column) as well as the topological distribution of coupling strengths (right column) also within (upper maps) and between (lower maps) the brains of the two guitarists at the three representative time points (panel G). Figures 3, 4 display the coupling strengths of the 10-s segment for FC1 (i.e., 1.25 Hz) in the two guitar duos, respectively. Results of coupling strengths for the other FCs (i.e., 2.5, 5, and 10 Hz) of these musical sequences of the two duos can be found in Supplementary Figures 1–6. The auditory representation of the 10-s segments analyzed here and the corresponding visualization of coupling strengths in real time can also be found in Supplementary Movies 1, 2. Please note that the soundtracks used for the coupling analyses described here were reconstructed from microphone records stored on the EEG computer with a sampling rate of 5,000 Hz. Notwithstanding this low sampling frequency, the auditory signals return the guitar tones well (Supplementary Movies 1, 2).

### Coupling Dynamics During a 10-s Segment in Duo 1

As can be seen in Figure 3, during the first time period (3.0 s) the communication between the guitars was realized through the connections coming from guitar A, which also sent strong connections to the right-temporal regions of guitarist A's brain. The brain of guitarist A also communicated with both guitars, while guitarist B's brain was connected to both guitars as well as guitarist A's brain. The strengths within the brain of guitarist A were predominantly strongest at parieto-occipital and frontal regions, and at parieto-occipital and left-temporal regions within the brain of guitarist B. The strongest between-brain strengths predominantly came from right-temporal regions of guitarist B's brain. Modularity analysis of the extended hyper-brain network including both guitars and both brains revealed two modules: one module (blue) comprised guitarist A's brain (except for two left fronto-temporal nodes) and both guitars (except for the low-frequency node of the guitar B), the second module (red) correspondingly comprised guitarist B's brain together with two nodes from guitarist A's brain and one node from guitar B, mentioned above. During the next time period of 6.0 s, both guitars and both brains were synchronized with each other to some extent. Guitar A exhibited bidirectional connections with guitarist B's brain, while guitar B communicated with both brains, but especially guitarist B's. The within-brain connection strengths were asymmetric in the two brains with right-temporal (guitarist A) and left-temporal (guitarist B) location predominance, also in frontal and parieto-occipital regions. The between-brain connections were rather scarce and predominantly located in right-temporal regions in both brains. The modularity analysis also partitioned the extended hyper-brain network into two modules, with a blue module comprising guitarist A's brain (except for four right fronto-temporal nodes) and three nodes of guitar B, and a red module comprising all remaining nodes (see Figure 3 for details). Finally, at the end of the 10-s segment (9.5 s), both brains were under the strong influence of both guitars, and they also strongly communicated with their own guitars and each other. Within the brains, the strongest strengths of guitarist A were located predominantly in the parieto-occipital regions, while those of guitarist B were strongest in the fronto-central regions. The strongest between-brain connection strengths were densely located in parieto-occipital and left-temporal regions in both brains. This time modularity analysis revealed three modules. The biggest (red) comprised three nodes from guitar A, all nodes from guitar B, central and parieto-occipital nodes from guitarist A's brain, and occipito-parietal and left-temporal nodes from guitarist B's brain. The second module (blue) is restricted to guitarist B and comprised the remaining nodes from guitarist B's brain. The third module (green) comprised the fronto-temporal nodes from the guitarist A's brain, and also one node from guitar A.

### Coupling Dynamics During a 10-s Segment in Duo 2

As can be seen in Figure 4, during the time period of 3.0 s, both guitars showed strong connections to both brains (especially to guitarist B's brain) and to each other. These connections were mostly bidirectional going from and to the brains/guitars. The within-brain connections were strongest in guitarist A's brain, with strongest strengths predominantly located in fronto-temporal regions, while those in guitarist B's brain were located predominantly right-parietally. The between-brain strengths were strongest at the right and left temporal regions, for guitarist A and B, respectively. Modularity analysis of the extended hyper-brain network revealed two modules: one module (blue) comprised guitarist B's brain (except for three left fronto-temporal nodes) and both guitars, the second module (red) correspondingly comprised guitarist A's brain together with three nodes from guitarist B's brain, mentioned above. During the next time period of 6.0 s, both guitars also showed strong connections to both brains and to each other, with links going from guitar A to both brains and links from guitar B predominantly to guitarist B's brain. These connections were mostly unidirectional, going from guitars to brains (especially, from guitar B), indicating strong influence coming from guitars. The within-brain connections are strongest in both guitarists at the fronto-temporal regions, while the between-brain strengths are densely located at frontal regions in guitarist A's brain and at occipital and right-temporal regions in guitarist B's brain. The modularity analysis partitioned the extended hyper-brain network into three modules, with two modules (blue and red) comprising both guitarists' brains (with the blue module additionally comprising three nodes from guitar A), and a third module (green) comprising the remaining node from guitar A, all nodes from guitar B, and frontal, central as well as right-temporal nodes from guitarist B's brain (see Figure 4 for details). Finally, at the end of the 10-s segment (9.8 s), both guitars and both brains were synchronized with each other to some extent. Within the brains, the strengths of guitarist A were strongest predominantly in the frontal and temporal regions, while those of guitarist B were considerably reduced and occupy the frontal and occipital regions. The between-brain connection strengths were strongest in the right parieto-occipital regions in guitarist A's brain and in right-temporal and frontal regions in guitarist B's brain. The modularity analysis revealed three modules: one module (blue) comprised all nodes from guitarist A's brain and one fronto-temporal node from guitarist B's brain, the second module (red) comprised the left part and some right parieto-occipital nodes of guitarist B's brain, together with one node from guitar A and all nodes from guitar B, and the third module (green) comprised the remaining part of guitarist B's brain and three remaining nodes from guitar A.

The inspection of other frequency components in both segments (see Supplementary Figures 1–6) showed (i) different temporal dynamic changes of network strengths for these frequency components, and (ii) that the networks within the same time periods exhibit different connectivity and community structures. All this indicates a complex interplay between different frequency components and underlying networks of the guitarists and instruments when improvising freely. Furthermore, it can be seen that dynamic changes of strengths themselves are oscillatory in nature and can be considered as second-order oscillations (Müller et al., 2018b).

### Power Spectral Density (PSD) of Second-Order Oscillations

**Figure 5:**
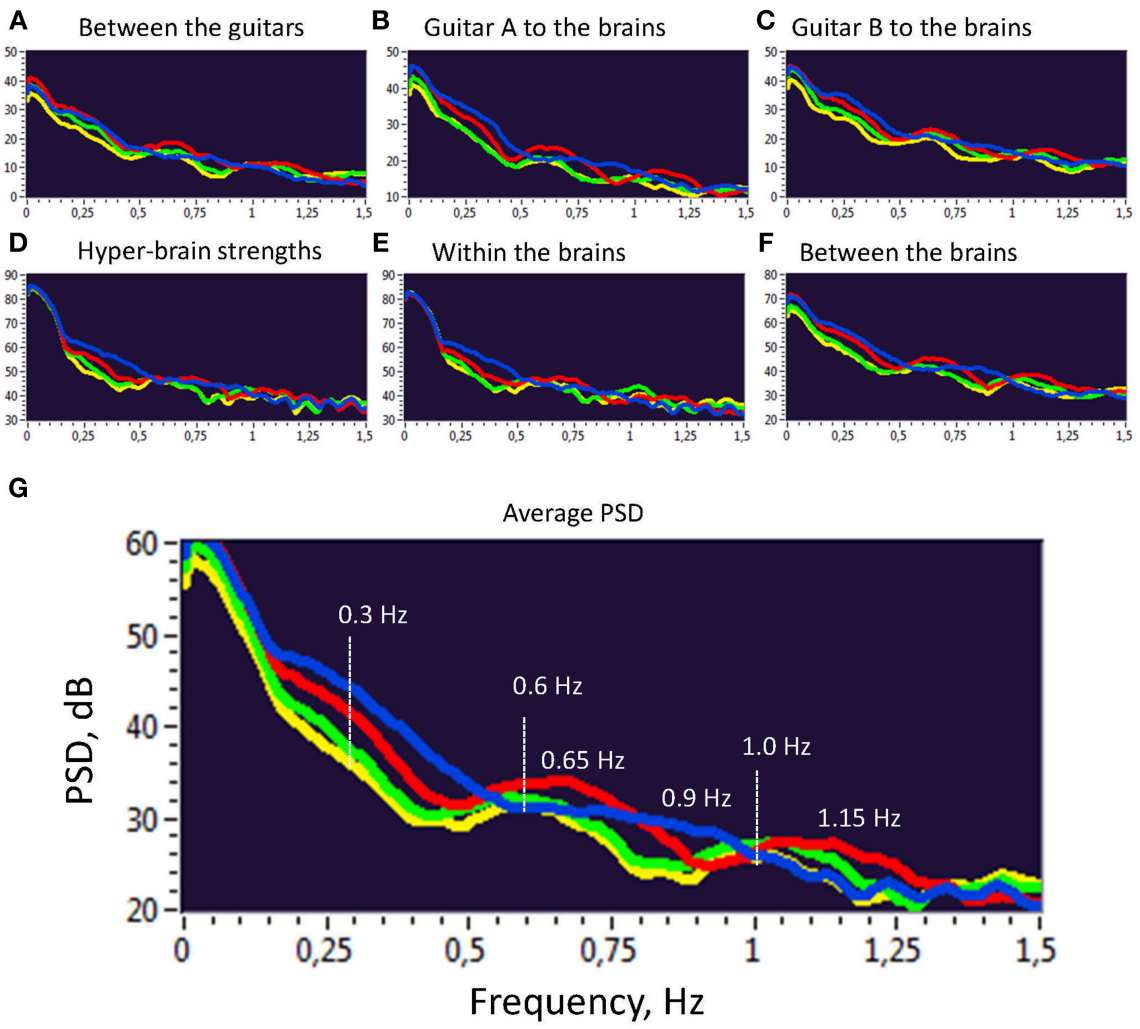
Power Spectral Density (PSD) of second order oscillations represented by strength dynamics. **(A)** PSD of coupling strengths between two guitars. **(B)** PSD of coupling strengths going from guitar A to the brains of both guitarists. **(C)** PSD of coupling strengths going from guitar B to the brains of both guitarists. **(D)** PSD of the hyper-brain coupling strengths. **(E)** PSD of the within-brain coupling strengths. **(F)** PSD of the between-brain coupling strengths. **(G)** Average PSD of all coupling strengths presented in A-F. For representation, PSD was averaged across 10 trails and two duos for the four FCs: FC1 (1.25 Hz), blue; FC2 (2.5 Hz), red; FC3 (5 Hz), green; and FC4 (10 Hz), yellow.

Further, we investigated the PSD of the second-order oscillations emerging through dynamic changes of coupling strengths between the instruments and brains as shown previously. To do so, we calculated the PSD across all 10 trials and 4 frequency components within the two duos for all the coupling strengths presented before. The PSD values were then averaged for the four frequency components separately. As can be seen in Figure 5, there are several PSD peaks in the frequency range between 0 and 1.5 Hz. Closer inspection of spectral peaks and the PSD course indicates that these peaks or frequency components can be divided into harmonics with a fixed frequency ratio (e.g., 1:2, 1:3, 1:4, etc.). This suggests that the coupling strengths between the instruments and the brains have a specific temporal structure, which apparently facilitate the free guitar improvisation and the underlying musical performance.

## Discussion

The primary objective of this study was to investigate the dynamic orchestration of brains and instruments during free guitar improvisation based on phase synchronization and extended hyper-brain network architecture including all the coupling types within and between the musicians' brains and guitars. The main findings are that such extended hyper-brain networks when playing guitar in duet (1) exhibit different temporal dynamic changes of network strengths, which are oscillatory in nature and have specific temporal structure for different EEG and instrument frequency components, and (2) feature different connectivity and community structures combining different brain regions and frequency components of instrument sounds, which share different, mostly heterogeneous modules (i.e., comprising different brains and instruments). It should be noted that the instrument's sound is a result of the musician's behavior, which is based on sensorimotor synchronization and action. At the same time, this sound influences the behavior of musicians through auditory sensory pathways and is in this sense an actor. In our view, music improvisation and interaction can be understood only when considering both bidirectional influences.

Previously, we have shown that both coupling strengths and community structures change their patterns depending on the oscillation frequency and musical situation (Müller et al., 2013, 2018b). We also pointed out that coupling strengths oscillate and exhibit so-called second-order oscillations, and that “the most important characteristic of the hyperbrain network organization is the existence of so-called hyperbrain modules sharing electrodes from two, three, or even four brains and characterized by strong connections or information flow within the modules and weak connections or information flow between the modules” (Müller et al., 2018b, p. 207). Here, we expand this observation to *extended* hyper-brain networks including instruments and their players' brains. It has been shown that modules or community structures can comprise one or two instruments as well as one or two brains, or different parts of them. Such attuned modular organization of extended hyper-brain networks provides efficient information flow within and between the brains and instruments (through behavioral control), and apparently supports the free improvisation when playing guitar in duet. The fact that modularity structure and coupling strengths dynamically change during playing indicates that brain-brain and brain-instrument interactions are never controlled by the same brain regions but rather alter regional dominances in accordance with the current musical situation and/or expectations. Even if it can be assumed that sensorimotor brain regions play an important role in music production, it is to be expected that fronto-parietal brain regions, which have to do with touch, theory of mind or intentions of others, together with the visual, auditory, and somatosensory cortices, are important teammates in music improvisation (Zatorre et al., 2007; Keller et al., 2014). It has been suggested that adaptive timing, motor sequencing, special organization of movements as well as anticipatory and attentional processes that enable rhythmic joint action and interaction are supported by distributed networks of cortical and subcortical brain regions (Zatorre et al., 2007; Keller et al., 2014). How all these functions and underlying cortical regions or brain structures are related to modular organization of extended hyper-brain networks must remain to be seen.

Recently, it has been reported that vocalizing patterns of singers are coupled to their respiratory and cardiac oscillations during choir singing (Müller et al., 2018a, 2019). Furthermore, the coupling between an instrument (piano assessed by MIDI tone onsets) and the brains of pianists performing a musical duet was investigated by using amplitude envelope correlations between EEG and MIDI signals (Zamm et al., 2018). In contrast, the method described here is based on the phase coupling between frequency components of amplitude variation in acoustic signals measured directly from the guitar and frequency components of raw EEG signals. In our view, this method provides more options to investigate the coupling between the instruments and the brains and also offers more information about behavioral and brain synchrony, especially when integrated into *extended* hyper-brain networks. We also used a directed coupling measure, which indicates the direction of the phase difference shift between two signals or refers to the preceding phase of one of the two signals. The preceding phase of one signal related to another can be understood two fold: (i) one signal influences the other or (ii) anticipation is at work. Both these processes are obviously very important during music improvisation (Biasutti and Frezza, 2009; Pecenka and Keller, 2011; Badino et al., 2014). In sum, this method provides high flexibility and accuracy in investigating different synchronization patterns between different instruments and musicians' brains when playing music in groups or assemblies.

In a number of studies, it has been shown that hyperscanning as a neuroimaging technique to investigate dynamic social interaction nowadays plays a crucial role in understanding the neural and physiological mechanisms of interacting or collective behavior (Dumas et al., 2011, 2014; Sänger et al., 2011; Keller et al., 2014; Acquadro et al., 2016; Müller et al., 2018a,b, 2019). Music performance has been considered a powerful catalyzer for social interaction (Keller et al., 2014; Acquadro et al., 2016). Extended hyper-brain networks including instrument-brain coupling would provide further insights into the interplay of music performance and underlying brain-body interactions. Moreover, it has been suggested that hyperscanning has great potential for music therapy (Hunt, 2015). Fachner et al. (2019) measured dual-EEG of an experienced therapist (“Guide”) and client (“Traveler”) in a real music therapy session, which was combined with audiovisual recordings. They identified and quantitatively investigated therapeutically important moments of interest (MOI) and no-interest (MONI). The authors suggested that combining dual-EEG (hyperscanning) with detailed audiovisual and qualitative data can provide pivotal information for further research into music therapy (Fachner et al., 2019). There is no doubt that registration of instrument-brain coupling would further improve this interesting and therapeutically significant approach.

In conclusion, this study shows that data acquisition and analysis methods for simultaneous EEG and instrument sound recordings from multiple persons are important for discovery of extended hyper-brain synchrony during interpersonal interactions. Synchronization patterns during free guitar improvisation assessed in terms of phase alignment for instrument–instrument, brain–brain, and instrument–brain interactions seem to reflect the complex interplay of different functions and underlying temporal dynamics of interpersonal coordination. These functions may include prediction of changed group behavior and executive control during group interaction. Future research needs to explore how these different functions assessed on the neuronal, behavioral, and group levels interact with each other and constitute mechanisms that establish and sustain interbrain oscillatory couplings in communication, voluntary action coordination, and social cognitive development. This method opens interesting perspectives for research on music interaction and may be an indispensable tool in the investigation of social interaction, music therapy, and rehabilitation dynamics.

## Data Availability

The datasets for this study will not be made publicly available because restrictions included in the consent statement that the participants of the study signed only allow the present data to be used for research purposes within the Max Planck Institute for Human Development in Berlin.

## Author Contributions

VM and UL designed the study and discussed the results, wrote the article, and read and approved the final version of the manuscript. VM acquired and analyzed the data.

### Conflict of Interest Statement

The authors declare that the research was conducted in the absence of any commercial or financial relationships that could be construed as a potential conflict of interest.
