# Supplementary material for: Dynamic Orchestration of Brains and Instruments During Free Guitar Improvisation
Source: Front Integr Neurosci. 2019 Sep 4;13:50. doi: 10.3389/fnint.2019.00050 (PMC6738335; doi:10.3389/fnint.2019.00050)
Supplement: Supplementary file 3 [file Data_Sheet_1.pdf]

Supplementary Material

1 Supplementary Data

Supplementary Movie 1. For duo 1 and FC1 (1.25 Hz), topological distribution of coupling strengths (left) and brain connectivity maps (right) within and between brains are presented. Diagrams on the bottom present: guitar traces obtained by microphone recording (guitar A, blue; guitar B, red), dynamic changes of coupling strengths within the brains of each of the two guitarists, dynamic changes of coupling strengths going from guitarist A's brain to guitarist B's brain (blue) and vice versa (red), dynamic changes of common coupling strengths, dynamic changes of coupling strengths going from guitar A to the guitar B (blue) and vice versa (red), dynamic changes of coupling strengths going from guitar A to the brains of both guitarists for each of the four frequency ranges of the guitar signal, which are indicated by color (low range, brown; middle range, cyan; high range, purple; whole range, yellow), dynamic changes of coupling strengths going from guitar B to the brains of both guitarists for each of the four frequency ranges of the guitar signal, and dynamic changes of coupling strengths going from guitar A (blue) and guitar B (red) to the brains of both guitarists averaged across the frequency ranges. Presentation occurs in real time. Please note that the sound tracks were reconstructed from microphone records stored on the EEG computer with a sampling rate of 5,000 Hz. Notwithstanding this low sampling frequency, the auditory signals return the guitar tones well.

Supplementary Movie 2. Duo 2 at the FC1 (1.25 Hz) is presented here. The representation is the same as in Movie 1.

2 Supplementary Figures

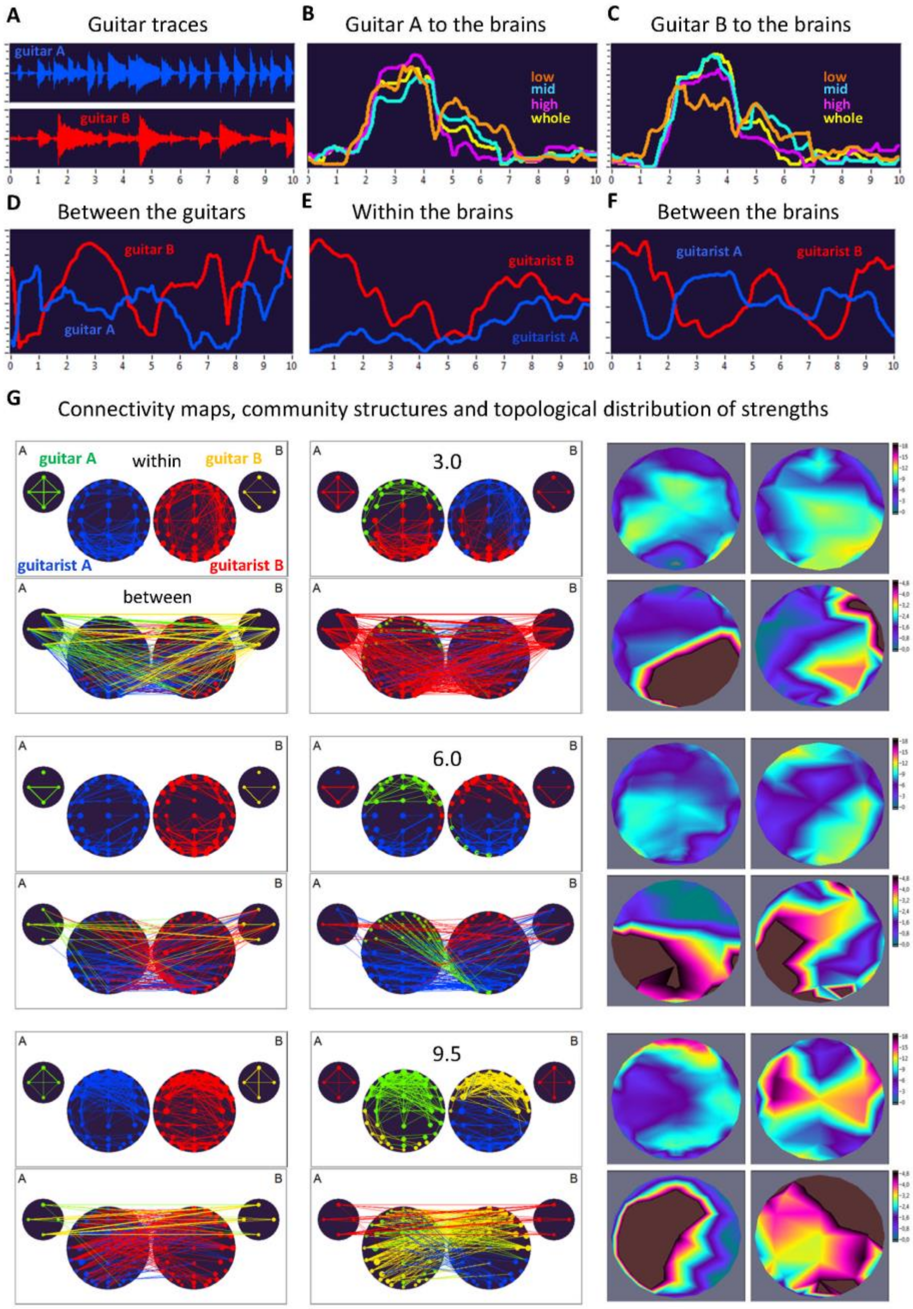

Supplementary Figure 1. Dynamic changes of strengths during a 10-s improvisation period for FC2 (2.5 Hz) in duo 1. (A) Guitar traces obtained by microphone recording: guitar A, blue; guitar B, red. **(B)** Dynamic changes of coupling strengths going from guitar A to the brains of both guitarists for each of the four frequency ranges of the guitar signal, which are indicated by color: low range, brown; middle range, cyan; high range, purple; whole range, yellow. **(C)** Dynamic changes of coupling strengths going from guitar A to the brains of both guitarists for each of the four frequency ranges of the guitar signal, which are indicated by color as in (B). **(D)** Dynamic changes of coupling strengths going from guitar A to the guitar B (blue) and vice versa (red). **(E)** Dynamic changes of coupling strengths within the brains of each of the two guitarists. **(F)** Dynamic changes of coupling strengths going from guitarist A's brain to guitarist B's brain (blue) and vice versa (red). **(G)** Brain connectivity maps, community structures, and topological distribution of coupling strengths. See Figure 2 for explanations.

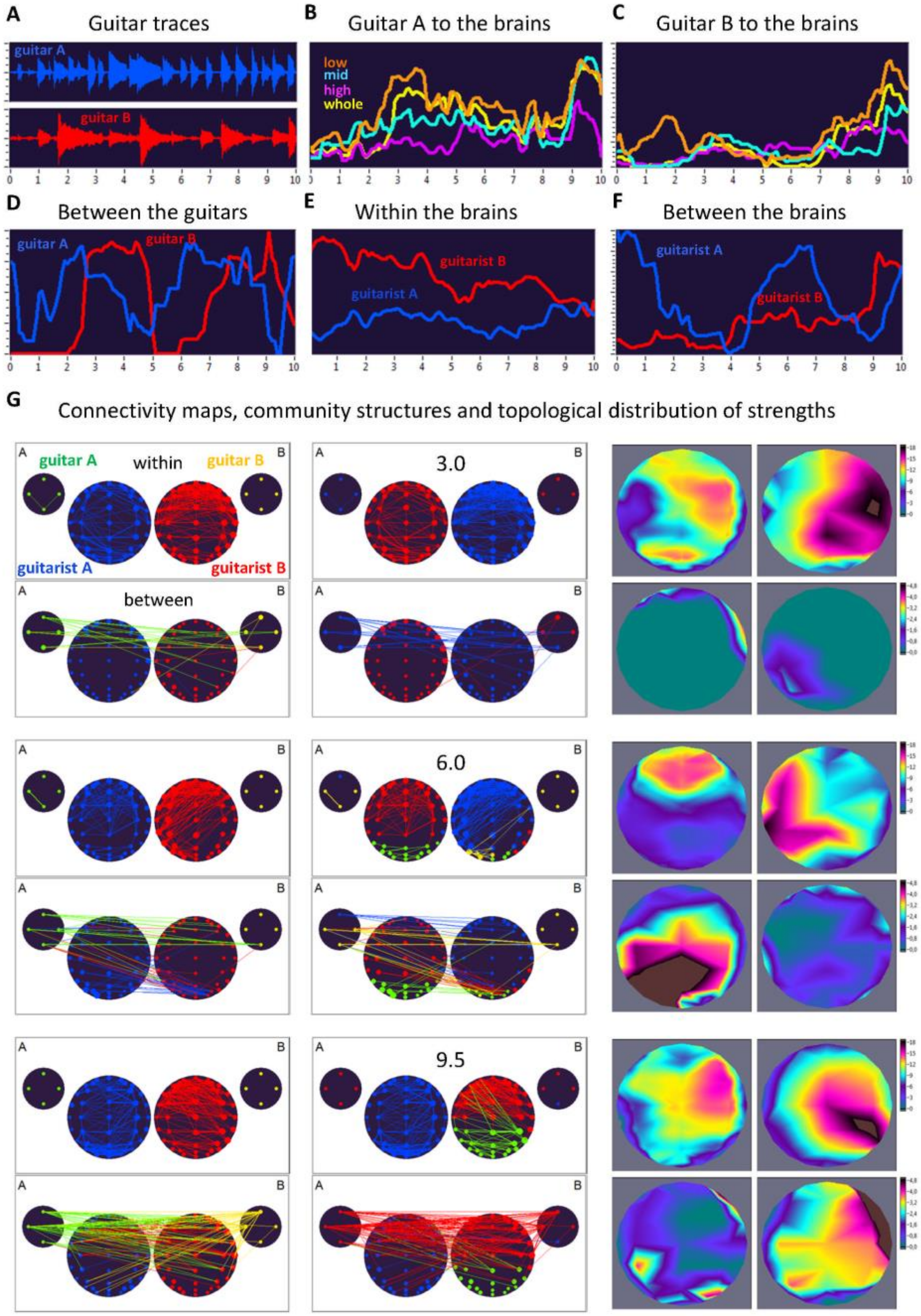

Supplementary Figure 2. Dynamic changes of strengths during a 10-s improvisation period for FC3 (5 Hz) in duo 1. (A) Guitar traces obtained by microphone recording: guitar A, blue; guitar B, red. **(B)** Dynamic changes of coupling strengths going from guitar A to the brains of both guitarists for each of the four frequency ranges of the guitar signal, which are indicated by color: low range, brown; middle range, cyan; high range, purple; whole range, yellow. **(C)** Dynamic changes of coupling strengths going from guitar A to the brains of both guitarists for each of the four frequency ranges of the guitar signal, which are indicated by color as in (B). **(D)** Dynamic changes of coupling strengths going from guitar A to the guitar B (blue) and vice versa (red). **(E)** Dynamic changes of coupling strengths within the brains of each of the two guitarists. **(F)** Dynamic changes of coupling strengths going from guitarist A's brain to guitarist B's brain (blue) and vice versa (red). **(G)** Brain connectivity maps, community structures, and topological distribution of coupling strengths. See Figure 2 for explanations.

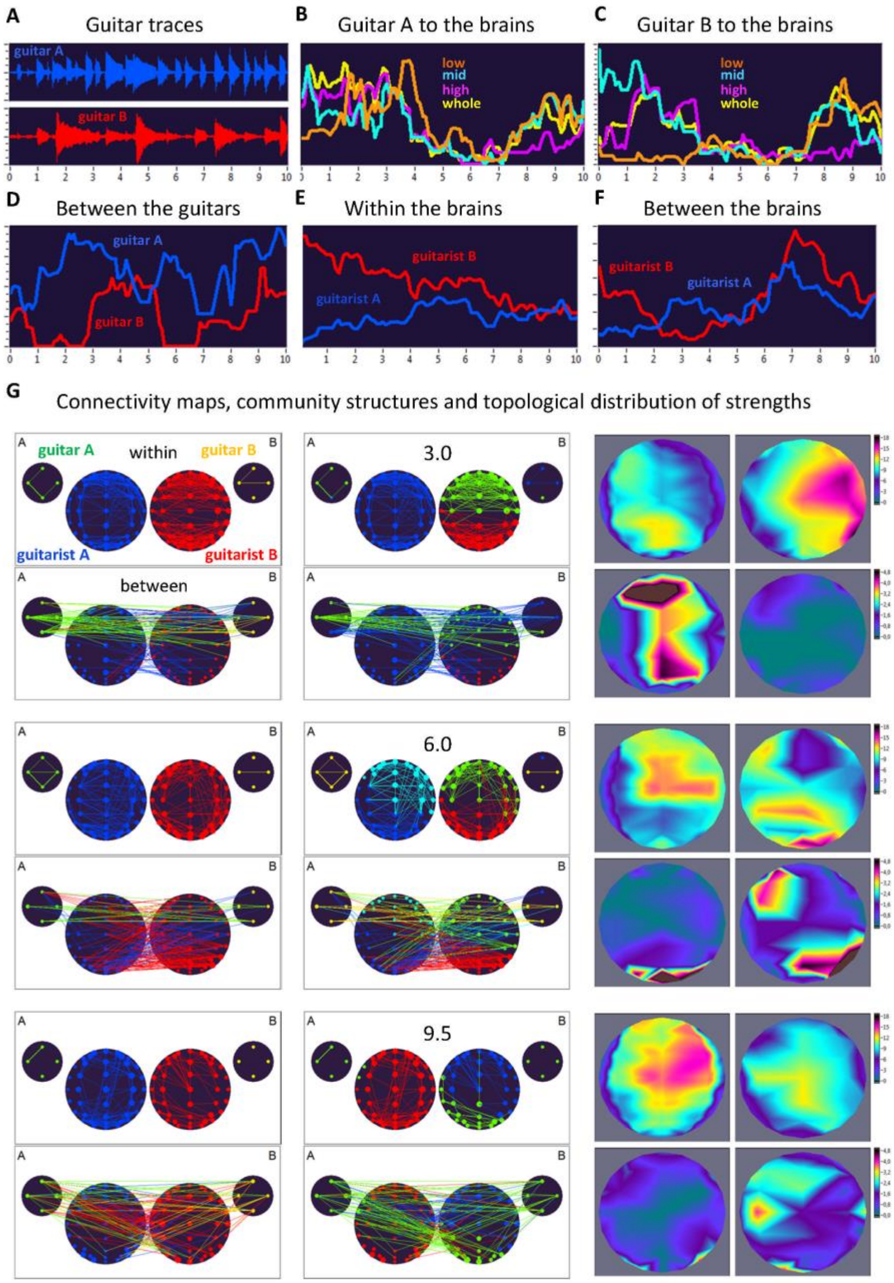

Supplementary Figure 3. Dynamic changes of strengths during a 10-s improvisation period for FC3 (10 Hz) in duo 1. (A) Guitar traces obtained by microphone recording: guitar A, blue; guitar B, red. **(B)** Dynamic changes of coupling strengths going from guitar A to the brains of both guitarists for each of the four frequency ranges of the guitar signal, which are indicated by color: low range, brown; middle range, cyan; high range, purple; whole range, yellow. **(C)** Dynamic changes of coupling strengths going from guitar A to the brains of both guitarists for each of the four frequency ranges of the guitar signal, which are indicated by color as in (B). **(D)** Dynamic changes of coupling strengths going from guitar A to the guitar B (blue) and vice versa (red). **(E)** Dynamic changes of coupling strengths within the brains of each of the two guitarists. **(F)** Dynamic changes of coupling strengths going from guitarist A's brain to guitarist B's brain (blue) and vice versa (red). **(G)** Brain connectivity maps, community structures, and topological distribution of coupling strengths. See Figure 2 for explanations.

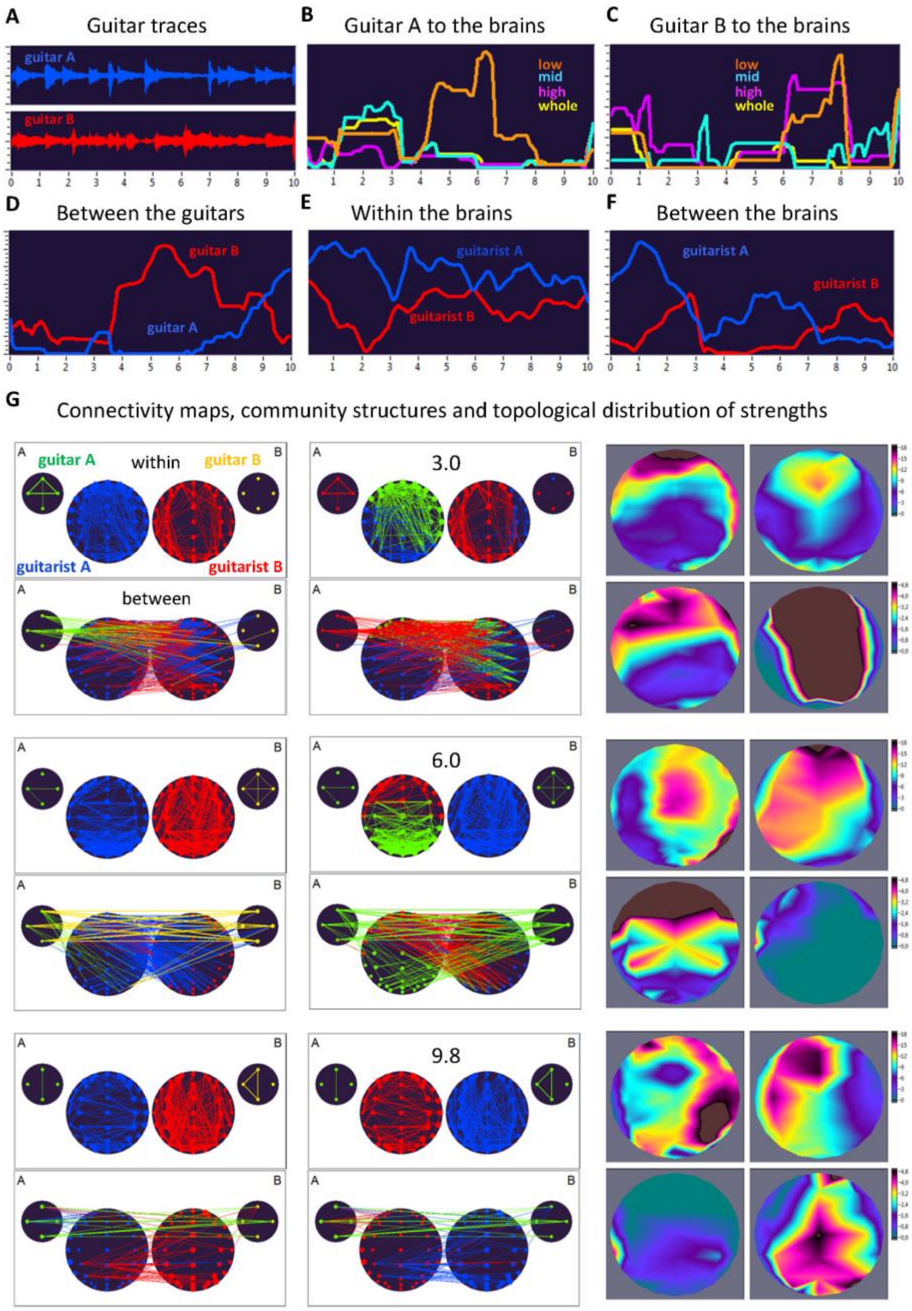

Supplementary Figure 4. Dynamic changes of strengths during a 10-s improvisation period for FC3 (2.5 Hz) in duo 2. (A) Guitar traces obtained by microphone recording: guitar A, blue; guitar B, red. **(B)** Dynamic changes of coupling strengths going from guitar A to the brains of both guitarists for each of the four frequency ranges of the guitar signal, which are indicated by color: low range, brown; middle range, cyan; high range, purple; whole range, yellow. **(C)** Dynamic changes of coupling strengths going from guitar A to the brains of both guitarists for each of the four frequency ranges of the guitar signal, which are indicated by color as in (B). **(D)** Dynamic changes of coupling strengths going from guitar A to the guitar B (blue) and vice versa (red). **(E)** Dynamic changes of coupling strengths within the brains of each of the two guitarists. **(F)** Dynamic changes of coupling strengths going from guitarist A's brain to guitarist B's brain (blue) and vice versa (red). **(G)** Brain connectivity maps, community structures, and topological distribution of coupling strengths. See Figure 2 for explanations.

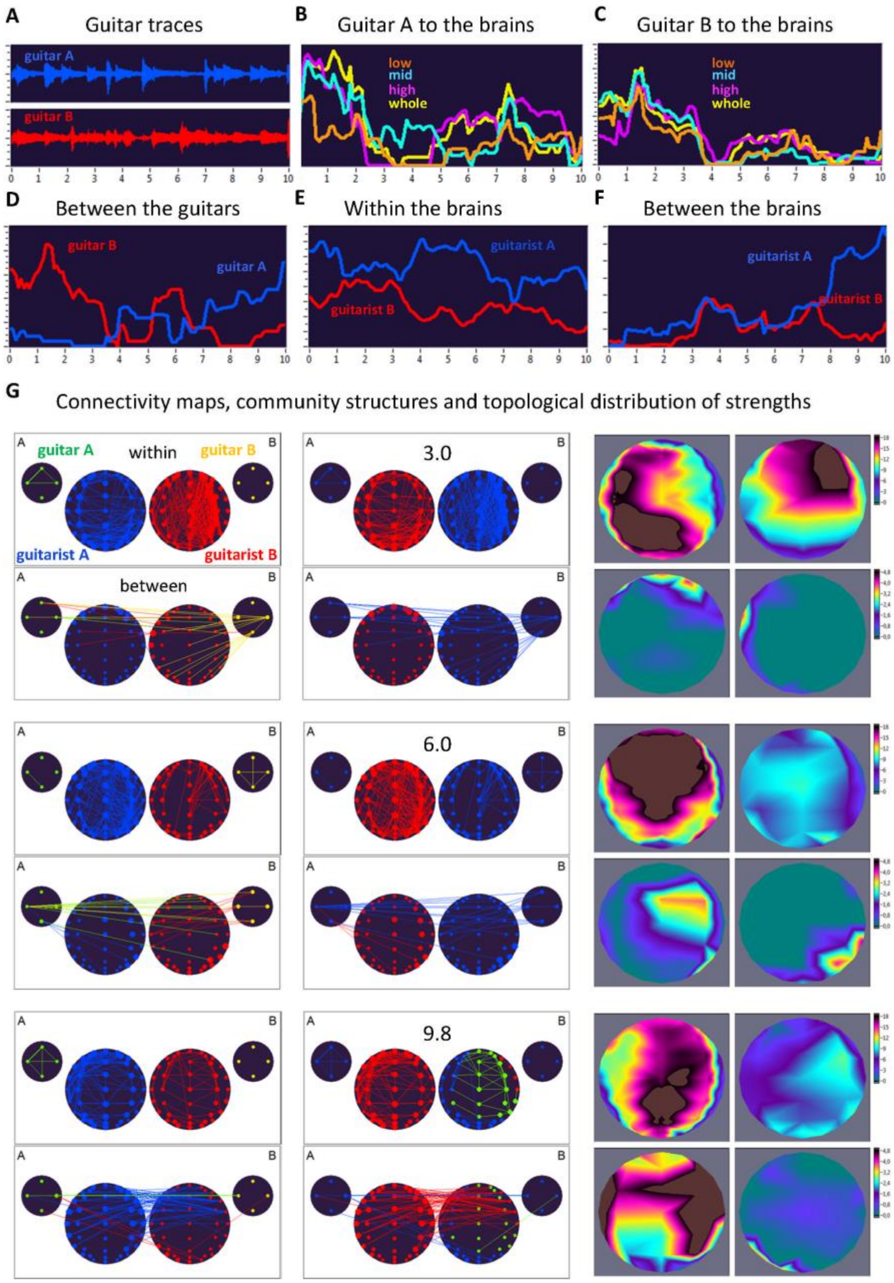

Supplementary Figure 5. Dynamic changes of strengths during a 10-s improvisation period for FC3 (5 Hz) in duo 2. (A) Guitar traces obtained by microphone recording: guitar A, blue; guitar B, red. **(B)** Dynamic changes of coupling strengths going from guitar A to the brains of both guitarists for each of the four frequency ranges of the guitar signal, which are indicated by color: low range, brown; middle range, cyan; high range, purple; whole range, yellow. **(C)** Dynamic changes of coupling strengths going from guitar A to the brains of both guitarists for each of the four frequency ranges of the guitar signal, which are indicated by color as in (B). **(D)** Dynamic changes of coupling strengths going from guitar A to the guitar B (blue) and vice versa (red). **(E)** Dynamic changes of coupling strengths within the brains of each of the two guitarists. **(F)** Dynamic changes of coupling strengths going from guitarist A's brain to guitarist B's brain (blue) and vice versa (red). **(G)** Brain connectivity maps, community structures, and topological distribution of coupling strengths. See Figure 2 for explanations.

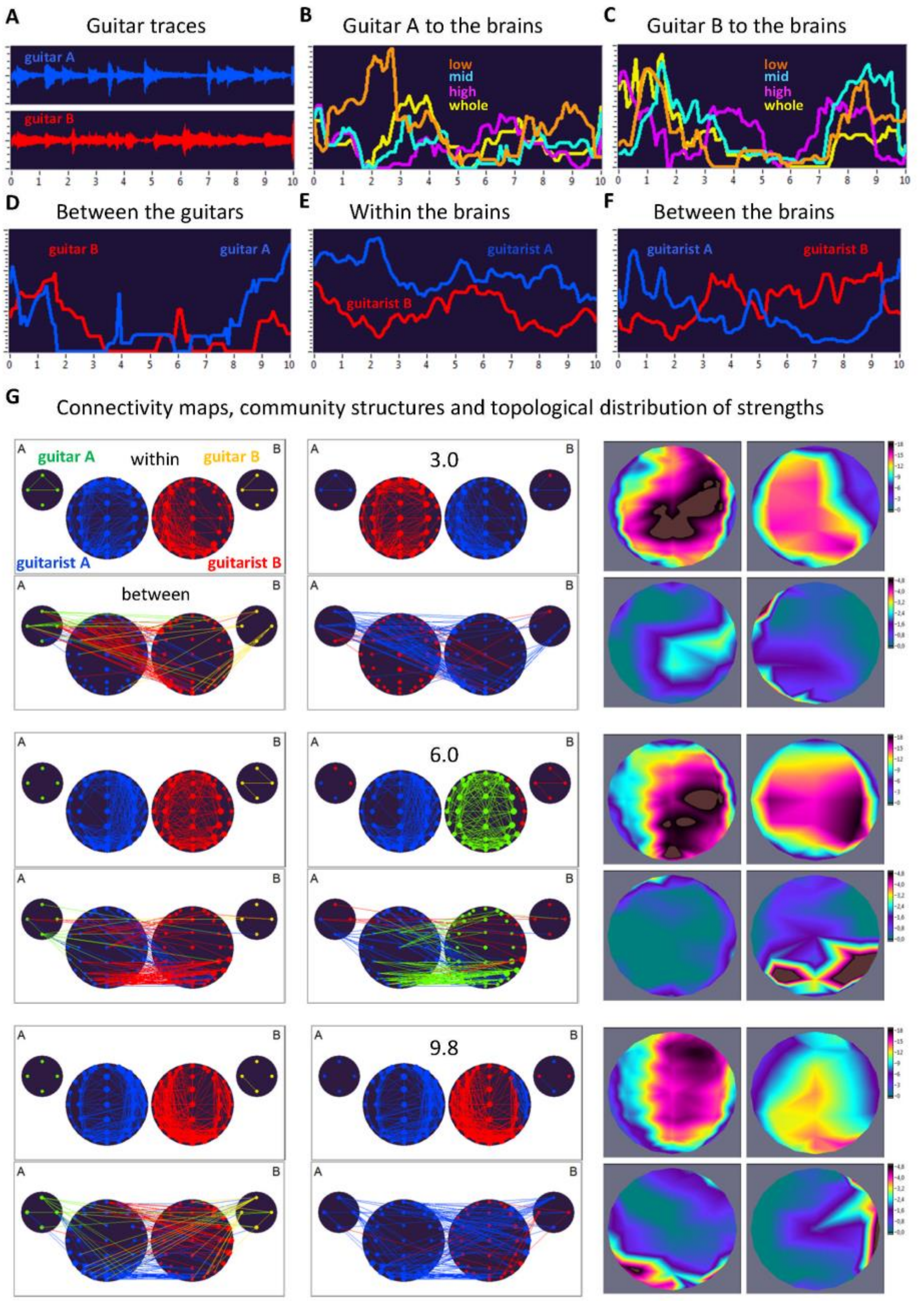

Supplementary Figure 6. Dynamic changes of strengths during a 10-s improvisation period for FC3 (10 Hz) in duo 2. (A) Guitar traces obtained by microphone recording: guitar A, blue; guitar B, red. **(B)** Dynamic changes of coupling strengths going from guitar A to the brains of both guitarists for each of the four frequency ranges of the guitar signal, which are indicated by color: low range, brown; middle range, cyan; high range, purple; whole range, yellow. **(C)** Dynamic changes of coupling strengths going from guitar A to the brains of both guitarists for each of the four frequency ranges of the guitar signal, which are indicated by color as in (B). **(D)** Dynamic changes of coupling strengths going from guitar A to the guitar B (blue) and vice versa (red). **(E)** Dynamic changes of coupling strengths within the brains of each of the two guitarists. **(F)** Dynamic changes of coupling strengths going from guitarist A's brain to guitarist B's brain (blue) and vice versa (red). **(G)** Brain connectivity maps, community structures, and topological distribution of coupling strengths. See Figure 2 for explanations.

MATLAB script for calculation of the Integrative Coupling Index (ICI)

```
function [PCI NCI ACI ICI12 ICI21] = DPcalcIC(Dphi,T,Dphi0)

% Müller V, Lindenberger U. 2011.
% Cardiac and respiratory patterns synchronize between persons during choir
% singing. PloS one. 6:e24893.
%
% Kitzbichler MG, Smith ML, Christensen SR, Bullmore E. 2009.
% Broadband criticality of human brain network synchronization.
% PLoS computational biology. 5:e1000314.
%
% %Inputs:
% %-Dphi: phase differences, Dphi~[-pi,pi], real numbers in a vector format
% %-Dphi0: phase difference threshold for synchronization Dphi~(0,pi/2)
% %-T: period in time points, positive, integer, 0<T<length(Dphi)
%
% %Outputs:
% %....The measures PCI, NCI, ACI, ICI, real numbers ~[0,1]
%
%
% %Data validation
%
% funcName = 'DP_PCI_NCI_ACI_ICI';
%
% varName='Dphi';
% testDPHI = { @(Dphi)isnumeric(Dphi),...
%              @(Dphi)isreal(Dphi),...
%              @(Dphi)min(Dphi)>=-pi,...
%              @(Dphi)max(Dphi)<=pi,...
%              @(Dphi)isvector(Dphi),...
%              @(Dphi)size(Dphi,2)==1 };
% param={{},{},{},{},{},{}};
% mode=['e','e','e','e','e','w'];
% execfun={{},{},{},{},{},@(Dphi)Dphi.'};
% default=nan;
% [~, Dphi] =
% DPvalidateData(Dphi,testDPHI,param,mode,execfun,default,varName,funcName);
%
Nphi = length(Dphi); %The total number of points
%
%
% varName='Dphi0';
% testDPHI0 = { @(Dphi0)isnumeric(Dphi0),...
%               @(Dphi0)isreal(Dphi0),...
%               @(Dphi0)Dphi0>=0,...
%               @(Dphi0)Dphi0<=pi/2,...
%               @(Dphi0)isscalar(Dphi0) };
% param={{},{},{},{},{}};
% mode=['e','e','e','e','e'];
% execfun={{},{},{},{},{}};
% default=pi/4;
% [~, Dphi0] =
DPvalidateData(Dphi0,testDPHI0,param,mode,execfun,default,varName,funcName);
%
%
% varName='T';
% testT = { @(T)isnumeric(T),...
```

```
%           @(T)isreal(T),...
%           @(T)T>0,...
%           @(T,Nphi)T<Nphi,...
%           @(T)round(T)==T,...
%           @(T)isscalar(T) };
% param={{},{},{},{Nphi},{},{}};
% mode=['e','e','e','e','e','e'];
% execfun={{},{},{},{},{},{}};
% default=nan;
% [~, T] =
DPvalidateData(T,testT,param,mode,execfun,default,varName,funcName);

% %Wrap phases in the interval (-pi pi)
% Dphi=mod(Dphi,2*pi);
% Dphi(Dphi>pi) = Dphi(Dphi>pi)-2*pi;

%If the time window is smaller than a period...
if Nphi<T
    %...adjust...
    T=Nphi;
    %...and print a warning message:
    cprintf('Magenta',['WARNING: Time window is smaller than one period.
Clearing period is adjusted to NwinLen=%d time points.\n'],T);
end

%Initialize the meters for...
Na=0; %...absolute sync points
Np=0; %...positive sync points
Nn=0; %...negative sync points

%Initialize the temporary meters for...
tempNa=0; %...absolute sync points
tempNp=0; %...positive sync points
tempNn=0; %...negative sync points

%Add a phase difference greater than the threshold at the end, so that we
%stop with a non sync point
Dphi = [Dphi;Dphi0+pi/10];

%Main loop
%For every point in the sequence...
for ii=1:Nphi+1;

    %...check if it is an absolute sync point
    if abs(Dphi(ii))<=Dphi0

        %...if yes, increase tempNa...
        tempNa=tempNa+1;

        %...and check if it is positive
        if Dphi(ii)>=0
            %...if yes, increase tempNp...
            tempNp=tempNp+1;
        else %...if not, increase tempNn...
            tempNn=tempNn+1;
        end
```

```
    else %...if it is not a sync point
        %...check if we have equal or more consecutive sync points than 1
period
        if tempNa>=T
            %...if yes, count the temporary sync points as sync points
            Na=Na+tempNa;
            Np=Np+tempNp;
            Nn=Nn+tempNn;
        end

        %...in any case, reset all temporary meters to 0
        tempNp=0;
        tempNn=0;
        tempNa=0;
    end
end

%Finally, calculate measures according to their definitions.
PCI = Np/Nphi;
NCI = Nn/Nphi;
ACI = Na/Nphi;
if ACI~=0
    ICI12 = ( (PCI+ACI)/(2*ACI) )*sqrt(PCI);
    ICI21 = ( (NCI+ACI)/(2*ACI) )*sqrt(NCI);
else
    ICI12=0;
    ICI21=0;
end
```
